# *Pseudomonas fuscovaginae* quorum sensing studies: 5% dominates cell-to-cell conversations

**DOI:** 10.1128/spectrum.04179-23

**Published:** 2024-03-21

**Authors:** Mihael Špacapan, Michael P. Myers, Luca Braga, Vittorio Venturi

**Affiliations:** 1 International Centre for Genetic Engineering and Biotechnology, Trieste, Italy; 2 African Genome Center, University Mohammed VI Polytechnic (UM6P), Ben Guerir, Morocco; Universita degli Studi Roma Tre Dipartimento di Scienze, Rome, Italy

**Keywords:** quorum sensing, cell-to-cell signaling, signaling, AHL, plant pathogens, intercellular, sociomicrobiology, phenotypic heterogeneity

## Abstract

A common feature of *N*-acyl-l-homoserine lactone (AHL) quorum-sensing (QS) systems is that the AHL signal is autoinducing. Once induced, a cell will further amplify the signal via a positive feedback loop. *Pseudomonas fuscovaginae* UPB0736 has two fully functional AHL QS systems, called PfsI/R and PfvI/R, which are inactive in a standard laboratory setting. In this work, we induce the QS systems with exogenous AHL signals and characterize the AHL signal amplification effect and QS activation dynamics at community and single-cell level. While the cognate signal is in both cases significantly further amplified to physiologically relevant levels, we observe only a limited response in terms of AHL synthase gene promoter activity. Additionally, the PfsI/R QS system exhibits a unique dramatic phenotypic heterogeneity, where only up to 5% of all cells amplify the signal further and are, thus, considered to be QS active.

**IMPORTANCE:**

Bacteria use *N*-acyl-l-homoserine lactone (AHL) quorum-sensing (QS) systems for population-wide phenotypic coordination. The QS configuration in *Pseudomonas fuscovaginae* is dramatically different from other model examples of AHL QS signaling and, thus, represents an important exception to the norm, which usually states that QS triggers population-wide phenotypic transitions in relation to cell density. We argue that the differences in QS dynamics of *P. fuscovaginae* highlight its different evolutionary purpose, which is ultimately dictated by the selective pressures of its natural habitat. We hope that this example will further expand our understanding of the complex and yet unknown QS-enabled sociomicrobiology. Furthermore, we argue that exemptions to the QS norm will be found in other plant-pathogenic bacterial strains that grow in similar environments and that molecularly similar QS systems do not necessarily share a similar evolutionary purpose; therefore, generalizations about bacterial cell-to-cell signaling systems function should be avoided.

## INTRODUCTION

Many bacteria use “Quorum Sensing” (QS) to coordinate phenotypes at a population level with extracellular auto-inducing molecules. QS, however, does not always correlate with cell density as the term would imply (1). *Pseudomonas* sp. QS systems often feature a LuxI-family synthase that can produce acyl-l-homoserine lactone (AHL) variants (2). LuxR-family transcriptional regulators (located in the cytoplasm) can dimerize with AHLs, bind to specific DNA motifs (Lux-boxes), and affect the transcription of target genes. The LuxI-family synthase gene promoter region usually features such a motif, which results in up-regulation creating an AHL positive feedback loop (3, 4). Therefore, the “QS” response is often quantified by measuring the increase in the AHL synthase gene transcription in response to the AHL signal. Plant pathogenic bacterial strains often use QS to coordinate virulence factor production, thus enabling “blitzkrieg” attack strategies, which involve a sharp transition to virulence-related behaviors at high cell densities (5). The *luxIR* QS genetic elements are genetically adjacent in the chromosome or plasmids, and in some cases, AHL QS systems feature an intergenic locus between the *luxI/R* genes, often implicated in their negative regulation (6). In many cases, AHL QS has been shown to regulate population-density-dependent behavior. However, assuming that this is a default feature of every AHL QS system is incorrect since in many cases it is influenced or even dominated by other physical, chemical, and biological parameters of the surrounding environment. In fact, considerable supra-regulation occurs as demonstrated in the two extensively studied hierarchically organized systems of *Pseudomonas aeruginosa*, as well as in AHL QS systems of other bacteria like *Vibrio harveyi, Ralstonia solanacearum*, *Rhizobium leguminosarum,* and *Agrobacterium tumefaciens* (1, 2, 7–9). Negative regulation, coupled to a positive feedback loop, can also result in phenotypic heterogeneity; therefore, QS could be one of the major factors promoting it (10, 11). QS could, therefore, also enable division of labor, specialization, and “bet-hedging” in bacteria (11). Therefore, AHL QS is not always a density sensor for social traits, and the generalist term “QS” with which we often refer to many bacterial cell-to-cell communication systems is misleading (11).

*Pseudomonas fuscovaginae* is one of the major causative agents of rice brown sheath rot and, during pathogenesis, constitutes the majority of the bacterial pathobiome (12). *P. fuscovaginae* UPB0736 (13) has two QS systems called PfsI/R and PfvI/R which are inactive, producing trace quantities of AHLs and displaying very low levels of transcription of the LuxI-family synthase genes when grown in laboratory conditions (14). Nevertheless, the LuxI family synthases PfsI and PfvI and the LuxR family regulators PfsR and PfvR are functional *in trans* in *Escherichia coli*. A transposon mutagenesis screen for AHL-producing strains has evidenced that *rsaM* (intergenically located between *pfsI* and *pfsR* genes) is involved in stringent QS repression (14). The exact mechanism of action of *rsaM* is currently unknown (15). Additionally, intergenically located between *pvfI* and *pvfR* there is the *rsaL* gene (14), which codes for a protein homologous to RsaL in *Pseudomonas aeruginosa* (16). Interrupting *rsaL* had no significant effect on *pfvI* promoter activity *in vivo*, while adding *rsaL, in trans* in *E. coli* with heterologous *pfvR* and exposed to exogenous AHLs decreased *pfvI* promoter activity (14). Studies on QS components *in trans,* however, may suggest that the QS receptors are more promiscuous than they biologically are (17).

In this article, we consider cells to be QS ON (or quorate) when the transcription of the cognate *luxI*-family AHL synthase is increased above the level of the wild-type baseline null expression. Considering that the two QS systems are also inactive at high cell density, we can conclude that QS activation does not correlate with cell density or significantly increase at a quorate cell density threshold. Despite the inactivity of the two QS systems in the laboratory, they play a role in rice pathogenicity *in planta* because the *luxI/R*-family gene knock-out mutants are attenuated in virulence (14). Therefore, it is assumed that AHL production and QS activation are activated *in vivo*. Additionally, it would be unusual for a bacterial species to conserve two QS systems if they did not have any role in overcoming selective pressures *in natura*. We, thus, hypothesize that the conditions needed for their activation are simply not being met when grown in standard laboratory conditions as opposed to when infecting rice plants. It was, therefore, decided to further characterize the activation/response dynamics of the two QS systems at a single-cell level by spiking liquid media with exogenous AHL signal. We reasoned that, due to the positive feedback loop, AHL saturation will mimic the environment in the immediate vicinity of a QS “activated cell” (18). This should enable us to describe the QS response dynamics despite not really knowing what the additional trigger for QS activation might be. However, this experiment is plausible only if the putative additional factor is wired alongside the AHL signal with an OR logic gate. Therefore, either the putative unknown stimulus or exogenous signal is needed to trigger a QS response and its potential positive feedback loop “domino effect”. If the additional factor is wired with an AND logic gate, no QS response to exogenous AHL should be observed.

## RESULTS

### The average QS transcriptional response of PfsI/R and PfvI/R to exogenous AHL concentration

**Fig 1:**
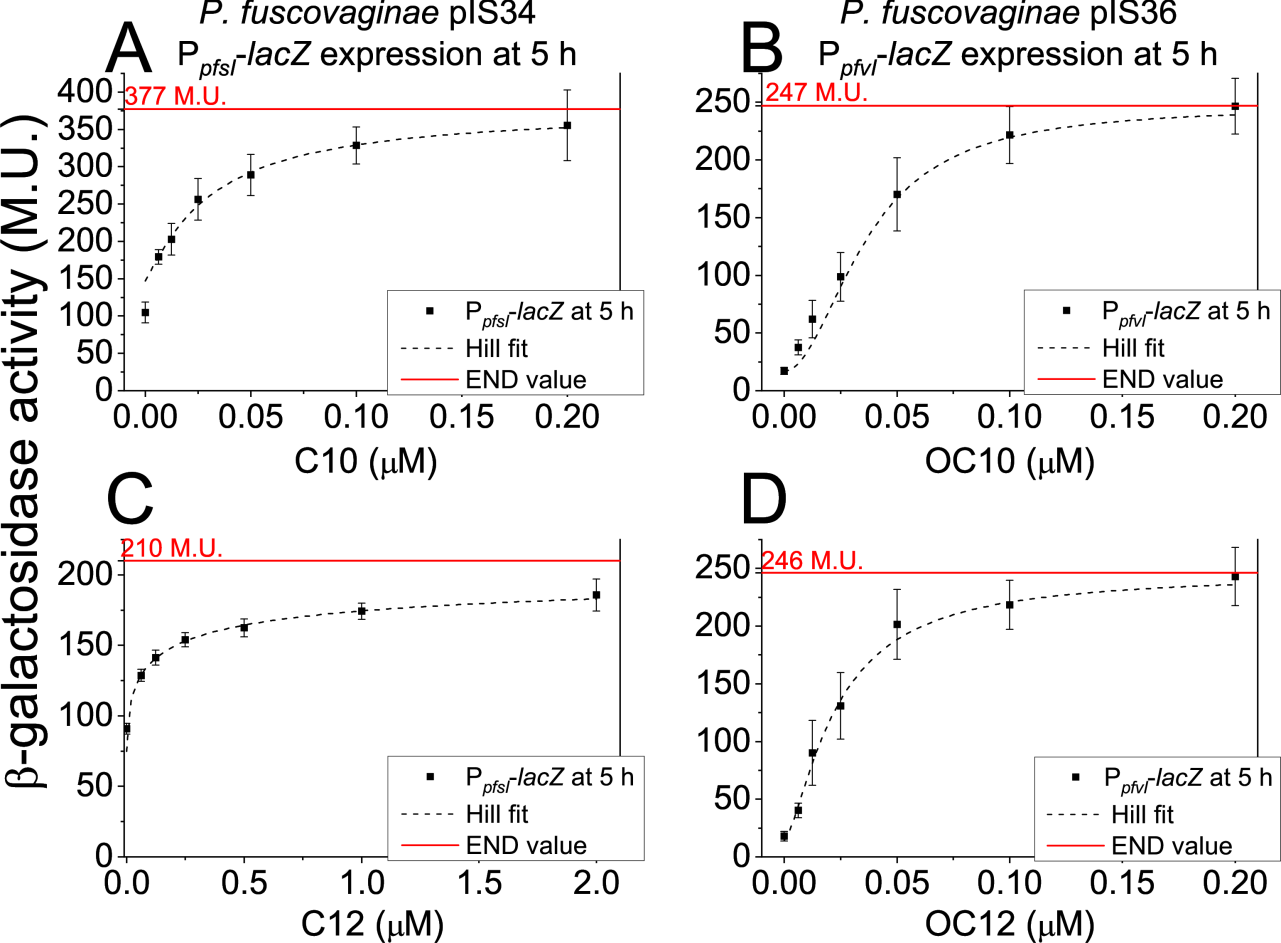
AHL dose-response of *P. fuscovaginae* QS. Beta-galactosidase activity of UPB0736 (pIS34) and UPB0736 (pIS36) after 5 h of incubation with shaking in liquid King’s B (KB) medium with exogenous AHLs. Data points indicate averages with error bars showing the standard error of means of at least three biological replicates. The dashed lines indicate a fitted Hill function (Hill1; OriginPro 8.5). The red line indicates the saturation value (END) of the Hill fit function. (**A**) C10 AHL dose-response of UPB0736 (pIS34), (B) OC10 AHL dose-response of UPB0736 (pIS36), (C) C12 AHL dose-response of UPB0736 (pIS34), and (D) OC12 AHL dose-response of UPB0736 (pIS36).

Since the PfsI/R and PfvI/R systems are inactive as they are repressed by RsaL and mostly by the RsaM under laboratory conditions [i.e., no detectable AHLs are produced (14)], it was initially of interest to determine their response to exogenously provided AHLs and be activated and/or respond to quorum concentration of AHLs. The growth dynamics of *P. fuscovaginae* are presented in Fig. S1, where we show the optical density at 600 nm (OD_600_) and colony-forming units (CFU) per milliliter after 5 and 24 h of incubation of a 1% (vol/vol) overnight culture inoculum with shaking. After 5 h, the strain reached values of approximately 1.5*10^9 CFU/mL. OD_600_ and CFU did not change much after that, so the culture is nearing the media carrying capacity. We then measured the QS response to exogenous AHLs of *P. fuscovaginae* strains carrying plasmids pIS34 (P*_pfsI_-lacZ*) and pIS36 (P*_pfvI_-lacZ*) assaying for promoter activity using the β-galactosidase Miller assay. Results evidenced that PfvR in *P. fuscovaginae* did not respond to a range of AHL varieties Fig. S2 to which it does respond *in trans* in *E. coli* (14). This is of no surprise, as of LuxR type receptors *in trans* can increase their promiscuity (17). As can be seen in Figure S2A a response was elicited via the PfsI/R system only by decanoyl-l-homoserine lactone (C10) and dodecanoyl-l-homoserine lactone (C12), therefore, their dose-response curve was determined. In Fig. 1A and C, we can see the QS response values, shown as β-galactosidase activity, of *P. fuscovaginae* UPB0736 (pIS34) after 5 h of growth when spiked with different concentrations of C10 and C12, respectively. All data sets of Fig. 1 are converging and could be fitted with a Hill function (dashed line) to estimate the QS response saturation. The PfsI/R system was more sensitive to C10 (Fig. 1A), compared to C12 (Fig. 1C). The culture had to be spiked with 10× more with C12 exogenous AHL to reach response saturation. Additionally, when spiked with C10 (Fig. 1A), *P. fuscovaginae* UPB0736 (pIS34) has a higher saturation value of 377 M.U. (red line) compared to when it was spiked with C12 (Fig. 1C), where the saturation value reaches 210 M.U.

With respect to the PfvI/R system, as shown in Fig. S2B *P. fuscovaginae* UPB0736 (pIS36) did respond to OC10 (Fig. 1B) and OC12 (Fig. 1C), which have a similar saturation value of cca. 250 M.U. *P. fuscovaginae* UPB0736 (pIS36) and (pIS34) exhibited similar AHL dose-response dynamics also after 24 h of growth (Fig. S3).

In summary, the PfsI/R system responded best to C10 (Fig. 1A) and with diminished sensitivity to C12 (Fig. 1C), while the PfvI/R QS system responded to OC10 (Fig. 1B) and OC12 (Fig. 1D) indiscriminately. Additionally, PfvI/R exhibited a small but significant response to C10 at 5 h (Fig. S4A) and 24 h (Fig. S4B) and a small response to C12 at 5 h (Fig. S4C) and 24 h (Fig. S4D).

### QS transcriptional response of PfsI/R and PfvI/R in *P. fuscovaginae rsaM* and *rsaL* null mutants

Since the *pfsI/R* and *pfvI/R* systems of *P. fuscovaginae* are stringently repressed by the RsaL and RsaM, it was of interest to determine the AHL response in the knock-out genomic mutants of these two repressors, i.e. with the AHL QS systems ON and how these respond to exogenous AHLs.

**Fig 2:**
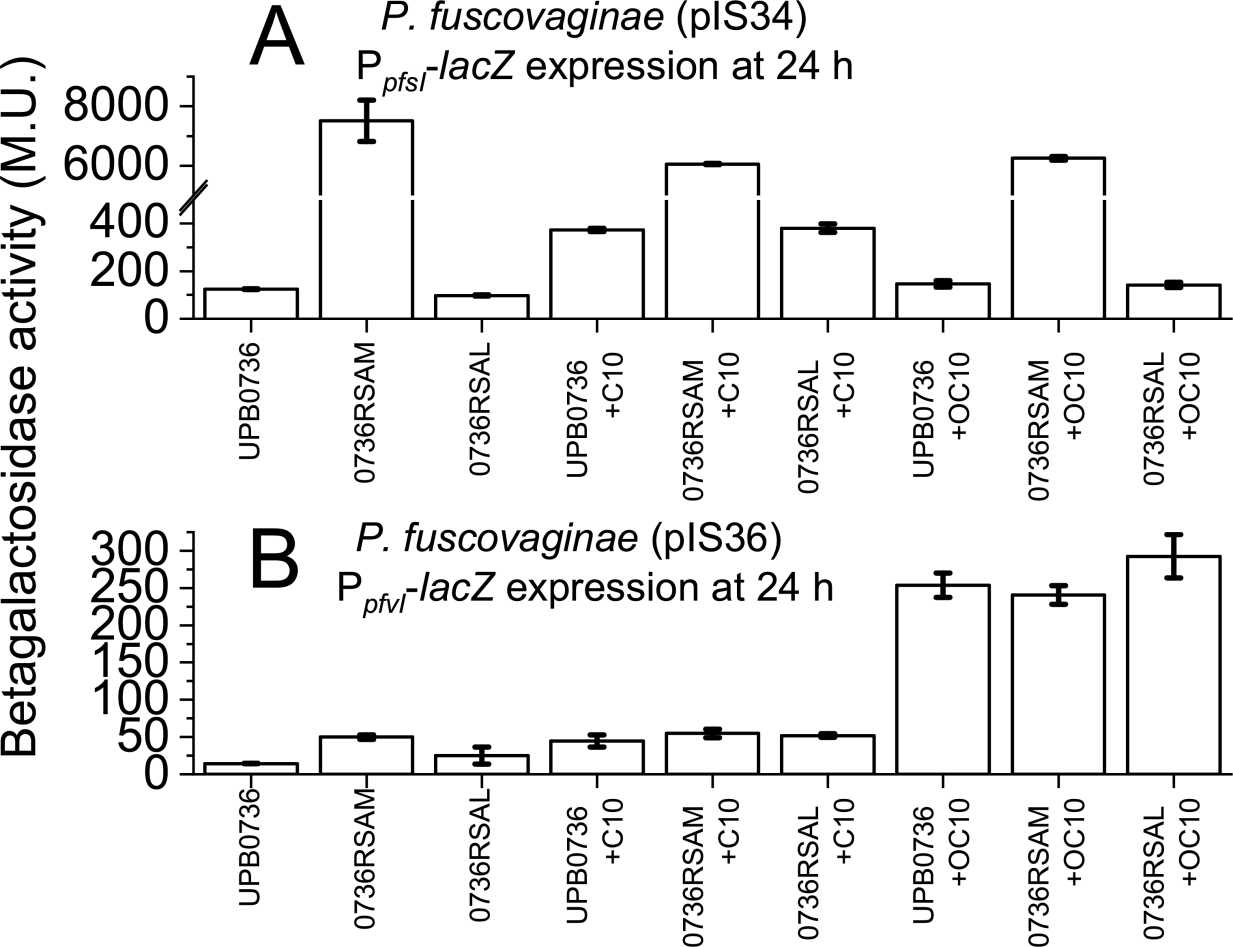
QS response of wild type relative to the *rsaM* and *rsaL* mutants with and without exogenous AHL. β-Galactosidase activity of UPB0736 (pIS34), UPB0736 (pIS36), 0736RSAM (pIS34), and 0736RSAL (pIS36) with and without exogenous AHL (C10 or OC10 at 0.2 µM). Strains were incubated with shaking in liquid KB media for 24 h. Data points indicate averages with error bars showing the standard error of means of at least three biological replicates. (**A**) β-Galactosidase activity of strains carrying pIS34, indicative of *pfsI* promoter activity. Please note that the *y*-axis is broken from 500 to 5,000 M.U. (**B**) β-Galactosidase activity of strains carrying pIS36, indicative of *pfvI* promoter activity.

Presented in Fig. 2A is the *pfsI* promoter gene promoter activity with and without C10 or OC10 (0.2 µM for both) in *P. fuscovaginae* UPB0736 (pIS34), 0736RSAM (pIS34), and 0736RSAL (pIS34) after 24 h. 0736RSAM exhibited very high *pfsI* promoter activity in all instances of approximately 7,000 M.U., which is considerably higher compared to the response observed in wild-type *P. fuscovaginae* UPB0736 with exogenously provided C10 at saturation levels (Fig. 1A). The PfsI/R response was not significantly increased in the *rsaL* mutant compared to the wild type (Fig. 2A). Additionally, the *rsaL* mutant was not more sensitive to exogenous AHLs in terms of the PfsI/R response (Fig. 2A), and C10 and OC10 did not alter the response in the *rsaM* mutant (Fig. 2A). In Fig. 2B, we present the *pfvI* gene promoter activity with and without C10 or OC10 in *P. fuscovaginae* UPB0736 (pIS36), 0736RSAM (pIS36), and 0736RSAL (pIS36) after 24 h. In this case, we see that *rsaL*, the putative repressor of the PfvI/R, does not affect *pfvI* promoter gene promoter activity, which is mostly dependent upon the addition of exogenous AHL. The PfvI/R response, however, was statistically higher in the *rsaM* mutant, relative to the wild type (Student *t*-test; *P*-value < 0.05) (Fig. 2B).

Overall, RsaM played a major role in the PfsI/R response, while RsaL played a negligible role in the PfvI/R response. RsaM slightly affected the PfvI/R response. Finally, the *rsaM* or *rsaL* mutations have no effect on the PfvI/R or PfsI/R response to C10 and OC10.

### AHL synthesis in response to AHL spiked media

**Fig 3:**
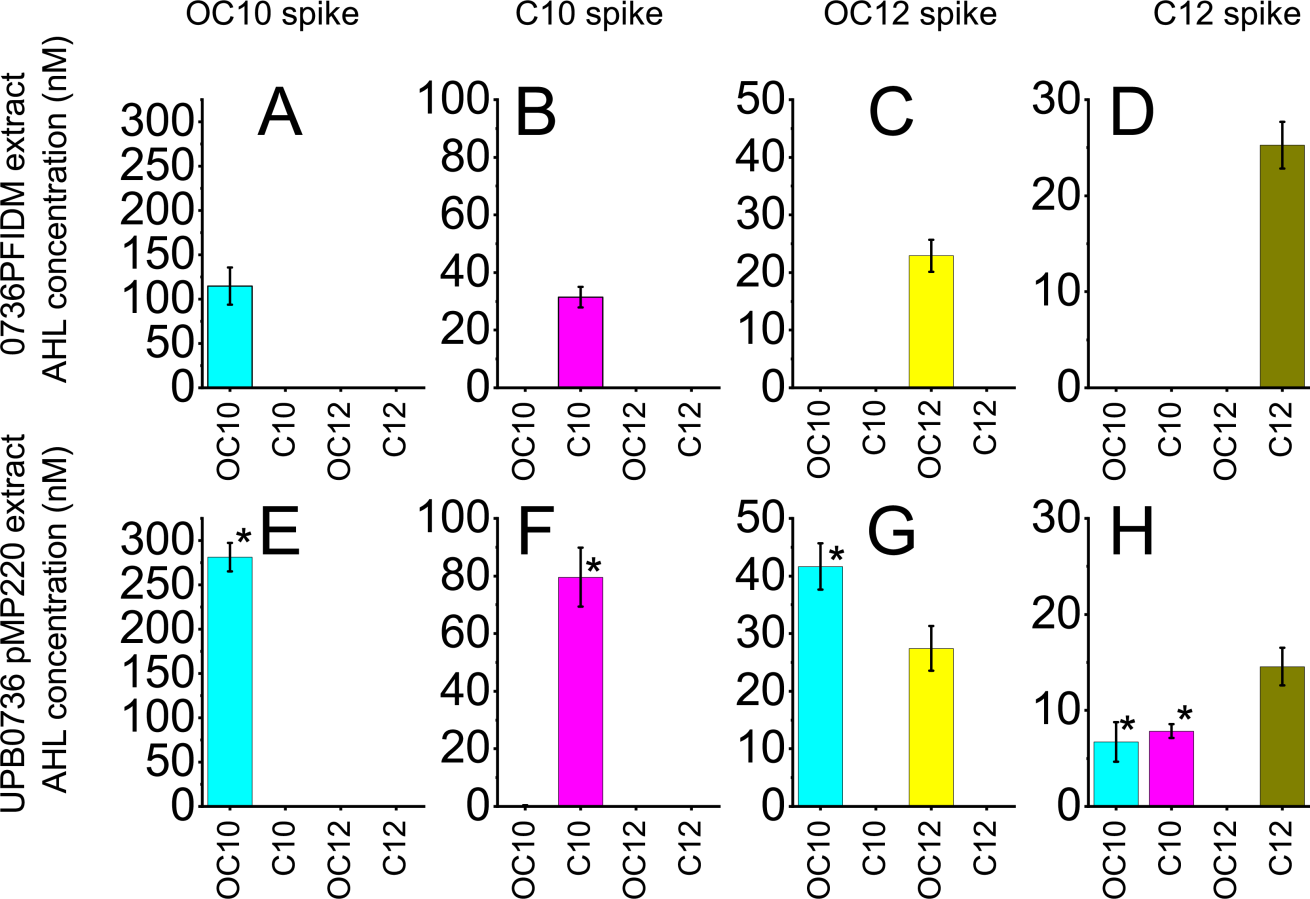
Exogenous AHL signal amplification after 24 h. On all graphs, we show the measured AHL quantities in organic solvent extracts of *P. fuscovaginae* incubated with exogenous AHLs in liquid KB medium with shaking for 24 h. Averages with error bars indicating the standard error of means are shown. Detected AHLs with averages lower than 5 nM are not shown. On (A)–(D), the 0736PFIDM double AHL synthase mutant was exposed to exogenous AHLs. On (E)–(H), the UPB0736 carrying an empty pMP220 vector as a control was exposed to exogenous AHLs. On (A) and (E), the media was spiked with OC10 up to 0.2 μM. On (B) and (F), the media was spiked with C10 up to 0.2 μM. On (C) and (G), the media was spiked with OC12 up to 0.2 µM. On (D) and (H), the media was spiked with C12 up to 2 µM. On (E)–(H), * denotes that the AHL quantity is significantly higher than the AHL quantity measured on (A)–(D), respectively (Student’s *t*-test; *P* ≤ 0.05).

To further characterize the PfsI/R and PfvI/R QS response, we also determined the levels of AHLs produced after 24 h in media spiked with exogenous AHLs. The double *pfsI/pfvI* AHL synthase knock-out mutant*, P. fuscovaginae* 0736PFIDM, was grown in four conditions, each supplemented with a structurally different exogenous AHL (i.e., 0.2 µM of C10, OC10, or OC12 and 2 µM for C12), and after 24 h, AHLs were extracted from the spent media and quantified as explained in the Materials and Methods (Fig. 3A through D). *P. fuscovaginae* 0736PFIDM did not produce any additional AHLs in response to the AHL spike, unsurprisingly since both *pfsI* and *pfvI* are inactivated in this strain and served as a control to estimate AHL amplification due to the QS positive feedback loop. Wild-type *P. fuscovaginae* UPB0736 was also grown under identical conditions, and after 24 h, AHLs were extracted and quantified (Fig. 3E through H).

In Fig. 3A, we can see that when the medium growing the double *pfsI/pfvI* null mutant was spiked with OC10, the concentration of extracted and measured OC10 was significantly lower compared to the concentration of OC10 when extracted from the spent supernatant of the wild-type strain (Fig. 3E). In Fig. 3B, we can see that when the medium was spiked with C10, the concentration of recovered C10 was also lower than the amount recovered from wild-type UPB0736 (Fig. 3F). When the medium was spiked with OC12, the concentration of OC12 did not significantly increase in the wild type (Fig. 3G) compared to the double *pfsI/pfvI* null mutant (Fig. 3C). The concentration of OC10, however, increased in the wild type relative to the double AHL synthase mutant (Fig. 3G and C). Surprisingly, when the medium was spiked with C12, the amount of recovered C12 was not higher in the wild-type UPB0736 (Fig. 3H) compared to the double synthase mutant 0736PFIDM (Fig. 3D); however, the concentration of OC10 and C10 increased.

Overall, it is, therefore, concluded that even if the average transcriptional responses of the AHL synthase genes were relatively low (Fig. 1 and 2), the C10 and OC10 AHL levels significantly increased when spiked with exogenous AHLs (Fig. 3).

### QS transcriptional response at a single-cell level

**Fig 4:**
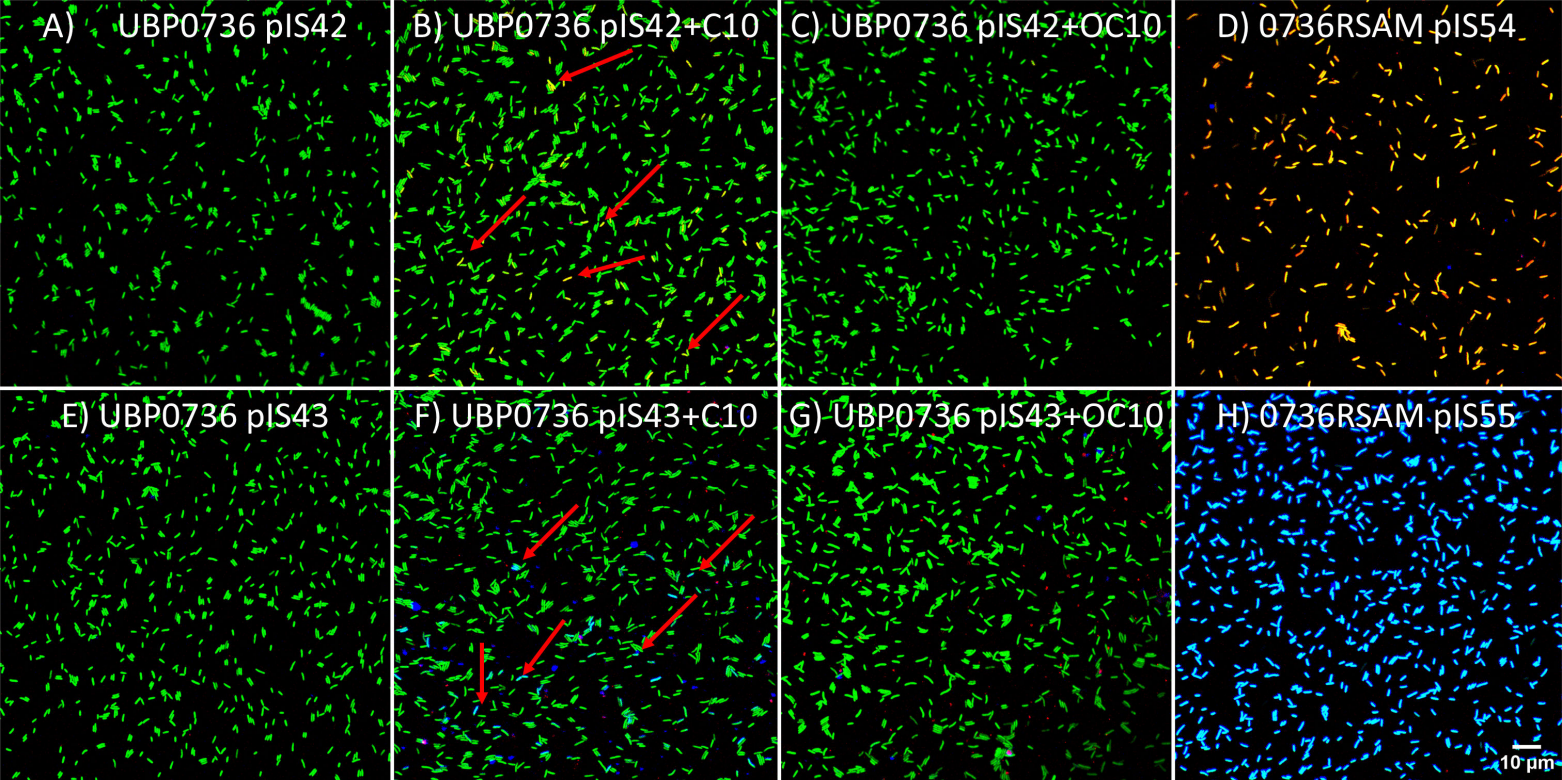
Distribution of QS “ON” cells after 24 h of growth. Bacteria were grown in liquid KB media with shaking, and after 24 h, cells were sampled and visualized with a confocal microscope. (**A**) UPB0736 (pIS42), (**B**) UPB0736 (pIS42) grown in 0.2 μM C10; red arrows indicate examples of P*_pfsI_* active cells, (C) UPB0736 (pIS42) grown in 0.2 μM OC10, (D) 0736RSAM (pIS54), (**E**) UPB0736 (pIS43), (F) UPB0736 (pIS43) grown in 0.2 μM C10; red arrows indicate examples of P*_pfsI_* active cells, (G) UPB0736 (pIS43) grown in 0.2 μM OC10, and (H) 0736RSAM (pIS55).

To obtain more information of the AHL QS response, we quantified at the single-cell level the activities of the *pfsI* and *pfvI* gene promoters in response to exogenous AHL spikes of C10 and OC10 at saturation concentrations. To do so, we grew *P. fuscovaginae* UPB0736 strains with fluorescent gene promoter transcriptional fusions growing with and without exogenous AHLs in shaken liquid media for 24 h and visualized single-cell fluorescence. A representative set of images is shown on Fig. 4. All the fluorescent reporter plasmid strains used (pIS42, pIS43, pIS54, and pIS55) have the *EGFP2* gene, coding for the green fluorescent protein, fused with the P*_km_* constitutive promoter (19). In pIS42 and pIS54, the *pfsI* promoter was fused to mCherry (red fluorescent protein) and the *pfvI* promoter was fused to *EBFP2* (blue fluorescent promoter), respectively. Whereas, in pIS43 and pIS55, the *pfvI* promoter was fused to mCherry (red fluorescent protein) and the *pfsI* promoter was fused to *EBFP2* (blue fluorescent promoter). The description and construction of all these plasmids are described in the Materials and Methods.

Following these experimental set-ups, in Fig. 4A through D, all bacterial cells exhibit green fluorescence, cells with an active *pfsI* promoter will be yellow (green and red), and cells with an active *pfvI* promoter should be cyan (blue and green). Whereas in Fig. 4E through H, cyan corresponds to cells with an active *pfsI* promoter and yellow corresponds to cells with an active *pfvI* promoter. In Fig. 4A, *P*. *fuscovaginae* (pIS42) is grown without exogenous AHLs, and no bacterial cells displayed yellow or cyan fluorescence, thus no cells had an active *pfsI* or *pfvI* promoter. In Fig. 4B, *P. fuscovaginae* UPB0736 (pIS42) was grown in the presence of 0.2 µM exogenous C10; it can be observed that some yellow bacterial cells appear, which indicated that *pfsI* promoter activity is highly heterogeneous in the bacterial population. Examples of such cells are indicated with red arrows. In Fig. 4C, *P. fuscovaginae* UPB0736 (pIS42) was grown in the presence of 0.2 µM exogenous OC10; surprisingly no cyan cells, which would correspond to *pfvI* promoter activity, were observed. In Fig. 4D, the *rsaM* mutant 0736RSAM (pIS42) was grown without the addition of exogenous AHLs and was observed that all cells were yellow, thus displaying high *pfsI* promoter activity. In Fig. 4E through H, similar results were observed as in Fig. 4E through H, the only difference being that promoters controlling mCherry and EBFP2 were swapped.

The cell images, shown in Fig. 4, and all the other two biologically independent replicates (images not shown) were segmented into objects using BiofilmQ (20). For every object, the mean red or blue fluorescence per green fluorescence was calculated. In Fig. S5, the relative frequency counts (binned object counts/all counted objects) binned according to their red mean object fluorescence/green mean object fluorescence (Fig. S5A) are presented. Additionally, in Fig. S5B, we present the relative frequency counts binned according to their blue mean object fluorescence/green mean object fluorescence. In both cases (Fig. S5A and B), *pfsI* promoter activity was estimated. All 0736RSAM cells had an active *pfsI* promoter as expected since RsaM was not functional. The *pfsI* promoter was active, however, only in a very small proportion of cells (up to cca. 5%) where the medium was supplemented with C10 (Fig. S5A and B). In Fig. S5C and D, *pfvI* promoter activity was estimated; however, we could not quantify any significant *pfvI* promoter activity, even when the culture was spiked with OC10. This was unexpected since when using the same promoter fragment and measuring its activity in the bacterial community (see above), an increase in the average β-galactosidase activity in OC10-spiked medium was observed (Fig. 1B). Additionally, as depicted in Fig. 3A and E, it was estimated that spiking the media with OC10 increased the OC10 concentration by at least twofold (see above). Therefore, we concluded that the *pfvI* promoter activity was likely not phenotypically heterogeneous but active in every cell at a low level, which is below the detection threshold of our promoter transcriptional auto-fluorescent fusion protein reporters. Finally, on Videos S1 to S8, we also grew UPB0736 (pIS42) and UPB0736 (pIS43) under solid agarose disks for 48 h in a 96-microtiter plate and visualized the fluorescence corresponding to *pfsI* promoter activity. After ca. 36 h both UPB0736 (pIS42) and UPB0736 (pIS43) exhibited red and blue fluorescence and from the patterns of fluorescence, it was evident that *pfsI* promoter activity was not uniform across the biofilm.

## DISCUSSION

### The hypothetical factor/stimulus-activating QS *in natura* is most likely wired alongside AHL signals with an OR logic gate

The existence of two fully functional quorum sensing systems in *P. fuscovaginae* UPB0736 (13), which are inactive when grown in standard laboratory conditions, raised many questions about their role *in natura* (11). Even if silent in the laboratory, they are important in rice plant pathogenesis *in vivo* (14). The rationale of the presented experiments was to assess the QS response dynamics when induced with exogenous AHLs to shed some light on how “QS” ON cells would behave. We reasoned that the vicinity of a QS cell, triggered with the unknown factor/stimulus, will be AHL saturated. Experiments were performed assuming that such a single cell will affect neighboring cells, as is assumed by default per definition of QS. It logically follows that we also assumed that the unknown QS activation factor/stimulus, which triggers QS ON, is wired to the QS response machinery alongside the AHL signal with an OR logic gate. Considering we did observe a response to exogenous AHL, we argue that this is the case. If the additional factor/stimulus is wired to the response with an AND logic gate, no response should be observed. We argue that our findings could help us understand how the QS ON cascade will spread in a population after local activation of the system. We believe that it is now timely to shift the way bacterial cell-to-cell communication systems are considered; for example, well-studied AHL QS systems have evidenced the presence of considerable supra-regulation. For a more in-depth discussion, we invite the reader to consider our recent perspective article (11), where this aspect of QS in bacteria is discussed at length. The additional factor/stimulus activating the QS system in *P. fuscovaginae* is currently to our knowledge unknown, and future research needs to address this (15).

Considering that a *rsaM* insertional mutant will have an overly active QS system (14), we suspect that RsaM is involved in either sensing this additional factor/stimulus or be the first “domino” that triggers the switch. Instead of being activated by an additional factor/stimulus, alternatively cells could transition ON due to stochasticity, and this would mean that QS regulation is in this case a generator of phenotypic heterogeneity.

### The average response has a relatively low value at saturation

As discussed above, *P. fuscovaginae* responds to exogenous AHLs and the response at signal saturation was unexpectedly low, considering that P*_pfsI_* promoter activity in the UPB0736 *rsaM::Tn5* mutant exhibits cca. 6,000–7,000 M.U. of activity (Fig. 2 and ref. 14). Existing data on *P. fuscovaginae* virulence has shown that inactivating either PfvR or PfsR results in virulence attenuation (thus implicating QS in pathogenicity), and interestingly, the *rsaM* mutant was also attenuated in virulence (14). This supports that the QS response cap is a feature for pathogenicity and not an artifact and that an overly zealous QS response is detrimental to pathogenicity. It could be that over-committing resources into the QS response has a negative physiological consequence for the cell, even if the QS response supports pathogenicity. Considering that no QS-related mutants were isolated in a random mutagenesis virulence screen (21) also supports this last hypothesis. Counter-pathogen strategies could involve saturating the environment with QS signal to trigger a pre-mature response (22). In this case, however, such a strategy would not be fruitful because the response is capped and will not reach the levels of a *rsaM* mutant. In *P. aeruginosa*, it has been shown that the QS cap often depends on the levels of LuxR- regulator (7). It cannot be excluded that a similar scenario occurs in *P. fuscovaginae* where *rsaM* may affect PfsR and PfvR levels.

The activity of the *pfsI* promoter saturated at higher levels and was 10 times more sensitive to C10 than C12 (Fig. 1A and C). The system appears to be tuned to primarily detect C10 and that C12 is sensed collaterally. In the case of *pfvI* promoter activity and OC10 and OC12, (Fig. 1B and D) we observed that the response saturates at similar level and the sensitivity of the QS system was identical, therefore *P. fuscovaginae* does not distinguish between these two AHLs. This could have biological significance, potentially due to inter-species signaling; however, considering that during pathogenesis and *in natura, P. fuscovaginae* constitutes most of the pathobiome (12), this might not be the case as it implies that during pathogenesis in rice *P. fuscovaginae* is prevalent and other sources of AHLs are less likely present.

### Production of AHLs is significantly increased in response to exogenous AHL

By determining AHL levels, it was evident that adding exogenous saturation levels (200 nM) of exogenous OC10 and C10 induced *P. fuscovaginae* to significantly synthesize AHLs (Fig. 3A, B, E and F) up to at least twofold. At 200 nM of either C10 or OC10, the response was saturated (Fig. 1A and B). We, therefore, concluded that the AHL concentration must have increased from 200 nM to cca. 400 nM, and this increase is high enough to be considered physiologically relevant. In other species using a similar cell-to-cell communication, AHL activates QS in a similar concentration range (23–27). This implies that the capped response shown in Fig. 1 is an evolutionary feature of the strain and that the response is functioning as evolutionarily intended. We, therefore, argue that this substantial AHL amplification has biological significance, even if the average AHL synthase gene transcriptional levels are relatively low.

Furthermore, we noticed that even if the PfsI/R system responds also to C12 (Fig. 1C) that when C12 is added exogenously, the strain will predominantly produce C10 as a response and C12 is not amplified (Fig. 3H). Similarly, even if the PfvI/R system responds to OC12 (Fig. 1D), only OC10 will be predominantly produced as a response (Fig. 3C and G). In both above cases, the amount of C10 and OC10 produced is relatively low, compared to the amount produced when also spiked with C10 and OC10. This indicates that PfsI/R has predominantly evolved for producing and sensing C10, while PfvI/R OC10. It would, therefore, be of interest to study if these AHLs confer a fitness advantage, which would give the observed promiscuity biological significance (11).

### PfsI/R QS is particularly phenotypically heterogeneous, and PfvI/R single-cell activity is below detection levels

Upon exogenous provision of saturated cognate AHL levels, the proportion of PfsI/R active cells in *P. fuscovaginae* is extremely low, whereas in the *rsaM* mutant, all the cells are QS active (Fig. 4). By counting individual cells (Fig. S5A and B), we estimated that the proportion of P*_pfsI_* active cells does not exceed approximately 5%; this rather extreme heterogeneity manifests at saturated AHL concentration and during very late growth phases (24 h). Considering that the AHL signals are nevertheless significantly amplified further makes us speculate that this heterogeneity could be an evolved feature that serves a specific, yet unknown evolutionary refined purpose *in natura*. In *P. aeruginosa*, it has been recently shown that only up to 80% of all cells will be quorate due to RsaL regulation (10) and that this phenotypic heterogeneity persists even when saturating concentrations of AHLs are added. The authors (10) raised the question of how the *rsaL* homologs might behave in other strains and wondered if other negative regulators with a different mode of action, like RsaM (15) would result in similar QS dynamics. The *rsaL* homolog, located between PfvI and PfvR, has been shown to inhibit *pfvI* promoter activity in *E. coli in trans* (14). In *P. fuscovaginae,* however, the *rsaL* mutant did not exhibit a significantly higher *pfvI* promoter activity (Fig. 2B, (14). Therefore, in *P. fuscovaginae* UPB0736, the function of *rsaL* remains to be determined. As for *rsaM*, when exogenously activated by provision of AHLs, the PfsI/R QS system is even more extremely heterogenic, where we estimate that only up to 5% of all cells are ON in a saturated AHL environment (Fig. S5A). This difference in heterogeneity in *P. fuscovaginae* and *P. aeruginosa* might be related to the different habitat of these strains. *P. aeruginosa* strains used in the experiments are descendants of an isolate originating from a human wound (28, 29), whereas *P. fuscovaginae* UPB0736 was isolated from diseased rice plants (13). This can lead to QS machinery with different activation dynamics because it is the selective pressure of the environment which dictates the evolution of QS systems (11). The phenotypes a QS system regulates are most likely strain specific, even if its components share some degree of mechanistical or molecular similarity, therefore, it is reasonable to assume the environment forces convergent evolution of different QS systems. Many instances of QS phenotypic heterogeneity have been described in strains which have a more similar habitat to *P. fuscovaginae*.

In *Sinorhizobium fredii*, a plant symbiotic organism, which also carries two QS systems, low amounts of AHLs increase the quorate population of cells for both QS systems (30). In the wild type, up to 80% of cells would be QS active, and unlike in our case, increasing and saturating AHL levels increase the percentage of ON cells to up to 100%. Interestingly, when adding root exudates, the percentage of QS ON cells would increase as well, indicating that the QS system is wired to an additional factor from the root exudate. Maybe, in a similar scenario, an additional factor/stimlus, also from the plant, could trigger RsaM mutant-like QS activation in *P. fuscovaginae*; future work will also focus on this direction.

In the plant pathogen *P. syringae*, the quorate cell proportion would represent 60% of all cells, and when the medium was AHL saturated, it reached a plateau of 80%. A similar observation was made for *Xanthmonas campestris*, another plant pathogen, which, however, has a different type of QS system (31). Considering that *P. fuscovaginae* UPB0736 is also a plant pathogen, we wonder if the differences in pathogenesis between strains can explain the dramatically lower proportion of QS ON cells observed here.

In a *P. putida* strain isolated from tomato rhizosphere, the AHL QS activation is heterogeneous when growing in a biofilm (32). While *P. fuscovaginae* UPB0736 is a plant pathogen, and unlike *P. putida,* was not originally isolated from the rhizosphere, it has been shown that its lipopeptides can also function as biocontrol agents (33). *P. fuscovagaine* strains have also been isolated from the plant rhizosphere where they could have plant growth-promoting ability (34, 35). *P. putida* heterogeneity persists even after saturating the media with AHL as well (32). When active, however, the population of ON cells represented up to 60% of the population. Videos S1 to S8 could suggest that if we quantified PfsI/R activation dynamics during biofilm growth, more cells would have a QS ON system in *P. fuscovaginae* as well as highlighting the importance of biofilms in plant-associated strains. In many of the above instances, phenotypic heterogeneity in terms of AHL signal promotion was often implicated in having a role in bet-hedging and division of labor. The PfsI/R QS could have a similar purpose; however, in this case, the division of labor and potential “bet hedging” would be even more extreme. The system could also serve a very specific, yet unknown purpose, for which such a phenotypic distribution cells is optimal.

In a previous publication, we argue that all QS cell-to-cell systems will inherently generate phenotypic heterogeneity (11), phenotypic homogeneity being one of its extreme outcomes. A limiting quantity of the QS signal receptor per cell might also explain the observed heterogeneity (23). It is important to note that this study employs plasmid-borne reporter constructs, and while all of them are low-copy plasmids (19, 36), the results shown might be affected by the introduction of 1–10 additional LuxR-binding sites, which drive the production of reporter fluorescent proteins or begatalactosidase activity. Future research efforts, therefore, should also be targeted at determining if there is a relationship between signal receptor concentration and the intensity of heterogeneity in the response.

At a single-cell level, we could not detect any PfvI/R significant increase in fluorescence with the *pvfI* promoter fusion constructs. The quantity of OC10 in OC10 spiked cultures, however, does increase up to 2-fold after 24 h (Fig. 3A, B, E and F). Additionally, by using the same promoter sequence, we could, with a Miller assay, measure a significant increase in the transcription of a liquid bacterial community of the *lacZ* gene in OC10 and OC12-spiked cultures (Fig. 1). We reason that we could not observe significant increase in *pfvI* promoter activity at a single-cell level (Fig. S5C and D) is due to our method not being sensitive enough. A characterization of PfvI/R QS activity at a single-cell level, therefore, remains to be determined.

### Phenotypic heterogeneity as a consequence of limited signal diffusibility

The hydrophobicity of AHLs will increase with the length of the acyl tail moiety, thus long-chain AHLs usually need additional mechanism to be more efficiently transported outside the cell since they do not dissolve as readily in watery environments (37–39). As a consequence, they tend to bind to glass and other plastic surfaces, and this makes them more difficult to recover. Additionally, it has been shown that long-chain AHLs, which increase in lipophilicity with length, interact with synthetic membranes (40). The 3-oxo modification of the acyl chain, however, will render the AHL more hydrophilic. OC10, the main signaling molecule of the PfvI/R system, is, therefore, much more hydrophilic than C10, the main signaling molecule of the PfsI/R system. The issues with limited diffusibility of lipophilic AHL in watery environments could be resolved with outer cellular vesicles (41), in which AHLs could be packed. In fact, it has been shown that the very hydrophobic C16 AHL signal of *Paracoccus denitrificans* is released mainly via membrane vesicles (42). It has also been reported that in *P. aeruginosa,* vesicles production is maximal at the transition from the exponential to stationary growth phase (43). Not all cells are likely to interact with a C10 AHL-packed membrane vesicle, thus enabling the phenotypic distribution of PfsI/R ON cells we observe in liquid media. Further research efforts should also focus more on this biochemical aspect of cell-to-cell signaling and their putative effect it could have on phenotypic heterogeneity.

### Cross sensitivity and the potential role of the double PfsI/R PfvI/R QS systems

In summary, the results presented here indicate that (i) both the PfsI/R and PfvI/R have a relatively low average response that is quickly saturated with AHLs; (ii) the PfsI/R system mainly responds to C10 and with decreased sensitivity to C12 and produces C10, while the PfvI/R system mainly responds to OC10 and OC12 but produces OC10; (iii) when the PfsI/R system exposed to saturating concentrations of AHL, only up to 5% of all cells are quorate and propagate the signal further.

What is the evolutionary purpose of having such a molecular mechanism in a pathogenic bacterium that causes rice sheath rot? We argue that points (i) and (ii) suggest that the QS system regulates a costly adaptive trait. While beneficial for the population, over-investing resources into it could be detrimental for the bacterial population economy. Capping the QS response might enable extreme bet-hedging strategies for very unlikely selective events, and division of labor might bring benefits due to increased specialization for a specific task. Since PfsI/R is active in only up to 5% of all cells might imply that those cells are performing a self-detrimental task which damages the individual but helps the population (e.g., non-specific antibiotic production/surfactant production).

Finally, the exact molecular mode of action of RsaM is also still unknown; understanding it could enable us to potentially exploit this genetic configuration that enables activation of up to 5% of all cells.

## MATERIALS AND METHODS

### Growth conditions

*Escherichia coli* strains were grown in LB low salt (LBLS; tryptone: 10 g/L, yeast extract 5 g/L, and NaCl 5 g/L) medium at 37°C with shaking and antibiotic selection when needed (100 mg/L for amp; ampicillin sodium salt, 20 mg/L for tetracycline hydrochloride; tet, 100 mg/L for kanamycin sulfate; kan, 20 mg/L for gentamicin sulfate; gm) *P. fuscovaginae* strains (Table 1) were grown in King’s B (KB) medium (10 g glycerol, 20 g Bacto proteose peptone N°3, 1.2 g of KH_2_PO_4_, and 1.5 g MgSO_4_ × 7H_2_O per liter of distilled water; equilibrated to pH 7 with NaOH and HCl) and antibiotic selection when needed (50 mg/L for nitrofurantoin; nf, 40 mg/L for tet, 100 mg/L for kan, 40 mg/L for g). Media were solidified by adding 1.5% (m/V) of agar when needed. For the β-galactosidase assay, spent media AHL extraction and single-cell fluorescence visualization, an overnight culture inoculated from solid media of *P. fuscovaginae* was grown in plastic sample tubes in 2 mL of medium at 30°C with shaking. Stock mixtures of AHLs dissolved in acetonitrile were added to growth media to complement with exogenous AHL when needed. Media with the same amount of acetonitrile was used as a control. For *in-vivo* fluorescent reporter visualization, a 1 µL droplet of 10-fold diluted overnight culture was placed on the bottom of a 96-well microtiter plate with a transparent bottom. To calculate the adjusted optical density at 600 nm, the culture was serially diluted twofold until the measured optical density value also decreased twofold. The total dilution was multiplied by the measured OD_600_ of the final dilution.

**TABLE 1:** *Pseudomonas fuscovaginae* UPB0736 strains

Strain and genotype	Reference
UPB0736 (wild type)	(13)
0736PFIDM (*pfvI*::*Tn5 pfsI*::*tet*)	(14)
0736RSAM (*rsaM*::*Tn5*)	(14)
0736RSAL (*rsaL::kan*)	(14)

### Strain construction

Conjugations with *P. fuscovaginae* strains were performed as described in reference (14) by using *E. coli* S-17 (44) in bi-parental conjugation or *E. coli* DH5α as the plasmid donor and *E. coli* pRK2013 (45) as the “Helper” strain in tri-parental conjugation. Nitrofurantoin or sometimes kanamycin was used to counter select for *E. coli* at concentrations indicated in the growth conditions for *P. fuscovaginae*. DNA was amplified with GoTaq (Promega), and restriction and ligation reactions (NEB, England) were performed per manufacturer’s instructions. To make pGS12 (Table 2), primers PM56 and PM57 (Table 3) were used with UPB0736 genomic DNA as a template. The fragment was TA ligated into pGEM-T easy (Promega) per manufacturer’s instructions. To make pGS13 (Table 2), primers PM54 and PM55 (Table 3) were used with UPB0736 genomic DNA and TA ligated into pGEM-T easy (Promega). To make pIS28, pGS12 (Table 2) was cut with BamHI-HF and SpeI-HF, and the insert was ligated into pRGC-BHR Pkm-gfp. To make pIS30, pGS13 (Table 2) was cut with EcoRI-HF, and the insert was ligated into pIS28 (Table 2) and checked for correct orientation. To make pIS34, pGS13 (Table 2) was cut with EcoRI-HF and ligated into pMP220 (Table 2) and checked for correct orientation. To make pIS36, pGS12 (Table 2) was cut with EcoRI-HF and ligated into pMP220 (Table 2) and checked for correct orientation. To make pIS39 (Table 2), primers PM72 and PM73 (Table 3) were used with pBAD-EBFP2 (Table 2) as a template. The fragment was digested with NotI-HF and SacI-HF and ligated into pmini-RGC (Table 2). To generate pIS40, pIS30 (Table 2) was cut with NotI-HF, and KpnI-HF and the insert was ligated into pIS39 (Table 2). To generate pIS41, pIS40 (Table 2) was cut with SpeI-HF and re-ligated into the same vector, and clones were subsequently checked for insertion with a reversed orientation. To generate pIS42, pIS40 (Table 2) was cut with SacI-HF and XhoI and ligated into pRGC-BHR (Table 2). To generate pIS43, pIS41 (Table 2) was cut with SacI-HF and XhoI and ligated into pRGC-BHR (Table 2). To generate pIS54 (Table 2), primers PM80 and PM81 (Table 3) were used with pmini-RGC (Table 2) as a template. The amplified fragment was digested with SacI-HF and inserted into pIS42 (Table 2). To generate pIS55 (Table 2), primers PM80 and PM81 (Table 3) were used with pmini-RGC (Table 2) as a template. The amplified fragment was digested with SacI-HF and inserted into pIS43 (Table 2). All constructs were verified with sequencing.

**TABLE 2:** Plasmids

Plasmid number	Relevant characteristic	Antibiotic	Reference
pMP220	empty lacZ reporter vector	tet	(36)
pRGC	empty fluorescence reporter vector	gm	(19)
pRGC-mini	empty fluorescence reporter vector	tet	(19)
pRGC-BHR	empty fluorescence reporter vector	gm	(19)
pRGC-BHR Pkm-gfp	P*_km_-EGFP2*	gm	(19)
pRGC-BHR Pkm3FP	P*_km_-EGFP2* P*_km_-mCherry* P*_km_-CFP*	gm	(19)
pRGC Pkm3FP	P*_km_-EGFP2* P*_km_-mCherry* P*_km_-CFP*	gm	(19)
pBAD-EBFP2	Vector with *EBFP2;* Addgene plasmid #14891	amp	(46)
pGS12	P*_pfvI_* from UPB0736	amp	This work
pGS13	P*_pfsI_* from UPB0736	amp	This work
pIS28	P*_km_-EGFP2* P*_pfvI_-CFP*	gm	This work
pIS30	P*_km_-EGFP2* P*_pfvI_-CFP* P*_pfsI_-mCherry*	gm	This work
pIS34	P*_pfsI_-lacZ*	tet	This work
pIS36	P*_pfvI_-lacZ*	tet	This work
pIS39	Promoterless *EBFP2*	tet	This work
pIS40	P*_km_-EGFP2* P*_pfvI_-CFP* P*_pfsI_-mCherry*	gm	This work
pIS41	P*_km_-EGFP2* P*_pfsI_-CFP* P*_pfvI_-mCherry*	gm	This work
pIS42	P*_km_-gfp* P*_pfsI_-mCherry* P*_pfvI_-EBFP2*	gm	This work
pIS43	P*_km_-gfp* P*_pfvI_-mCherry* P*_pfsI_-EBFP2*	gm	This work
pIS54	P*_km_-gfp* P*_pfsI_-mCherry* P*_pfvI_-EBFP2*	tet, gm	This work
pIS55	P*_km_-gfp* P*_pfvI_-mCherry* P*_pfsI_-EBFP2*	tet, gm	This work

**TABLE 3:** Primers

Primer	5′- > 3′ sequence
PM54	GTCGACTCCACACCAGCGTCCAATCCAG
PM55	GTCGTTTCAGCCTTATCTTGC
PM56	CCCACTAGTGCAGGCAGTTCTGATCAAAG
PM57	CCGGATCCTTCTCAGTCTGTCGGAGCCCA
PM72	GCGGCCGCAAAGAGGAGAAATTAAGCATGGTGAGCAAGGGCG
PM73	GAGCTCTTACTTGTACAGCTCGTCCAT
PM80	ATATGAGCTCTTCAAGAATTCTCATGTTTGACAGC
PM81	ATATGAGCTCTTCCATTCAGGTCGAGGTGGC

### Betagalactosidase transcriptional promoter activity assays

Miller assays were performed as described by reference (14). All the data shown represent averages of at least three independent biological replicates with the error bars indicating the standard error. The saturation limit was estimated by fitting the Hill1 function to measured data in OriginPro 8.5.

### Organic solvent AHL extraction

Cultures were centrifuged (8 kRCF 3 min) and the spent media was filtered (0.2 µm pore diameter). Six hundred microliters of spent media was acidified with 13 µL of 1 M formic acid. Then, spent media was mixed 1:1 with water-saturated ethyl acetate and shaken at room temperature for 30 min. Four hundered microliters of the organic phase was collected, completely evaporated after incubation at 95°C, and stored at 4°C until analysis.

### HPLC/MS-MS

Extracts were reconstituted into 100 µL of 0.1% formic acid in water. Ten microliters of the reconstituted extract was injected onto a 100 µm × 15 cm column packed with Intersustain AQ 3 µm beads (GL Sciences) and developed with a 5%–80% gradient of Acetonitrile in 0.1% formic acid in 20 min. The effluent of the column was directed into the orifice of a 6550 QTOF (Agilent). The QTOF was run in targeted ion mode with the quantitation based on the conversion of the precursor mass to the 102.o5 *m*/*z* product ion. The elution times of the AHLs were scouted using purified standards. The peaks were extracted, and the peak areas were determined using the MSHunter software package (Agilent). AHLs were quantified according to their peak area, relative to the peak area of samples with AHL standards.

### Single-cell fluorimetry

Two microliters of culture was placed on top of a glass coverslip, covered with a thin agarose pad, inverted, and sealed onto a glass objective. Cells were then imaged with a Zeis 880 Airyscan confocal microscope. Images were taken with a 63×/1.4 oil immersion objective. Images were taken at a 1,024 × 1,024 resolution with a 16 Bit pixel intensity depth. The first track for imaging red fluorescence was excited with a 543 nm laser, and the emission was cut off from 578 nm to 696 nm. The second track for imaging green fluorescence was excited with a 488 nm laser, and the emission was cut off from 493 nm to 590 nm. The third channel for blue fluorescence was excited with a 405 nm laser, and the emission was cut off from 371 nm to 451 nm. To visualize images, a composite projection was done in Fiji 1.53t (47). Red fluorescence was visualized from 500 to 5,000; green from 500 to 10,000; and blue from 500 to 1,000 pixel intensity units. Objects were counted with BiofilmQ (20), and cells were segmented according to their green fluorescence channel by using the default settings. Afterward mean object fluorescence of either channel 1 or 3 per mean object fluorescence of channel 2 was calculated. Objects were binned, and histograms were drawn in OriginPro8.5. For live imaging of cells growing on biofilms, 2 µL of 10× diluted culture was placed on a transparent bottom of a 96 well plate. A thin KB medium agar disk was placed on top of the culture. The 96 plate was sealed and incubated in the Operetta High Content analysis system (PerkinElmer) at room temperature for 48 h. A 40× objective was used in confocal mode with an mtagBFP filter (ex:390–420 nm em:430–500 nm) for blue fluorescence, Alexa 594 (ex: 530–560 nm em: 570–650 nm) for red fluorescence, and Alexa 488 (460–490 nm) for green fluorescence. Images were ordered in hyperstacks with Fiji 1.53t, Bleach Correction was applied, and the image was saved as an .avi file, using JPEG compression and 7 fps. Videos were uploaded on Youtube and are available on https://www.youtube.com/playlist?list=PLRxYiWDI2LqQW79NH1ncEpHhXA75-hgvJ.

## Data Availability

All raw data, strains, and other genetic constructs are available upon request.

## SUPPLEMENTAL MATERIAL

Supplemental material spectrum.04179-23-s0001.docx Figures S1 to S5 and Videos S1 to S8.

ASM does not own the copyrights to Supplemental Material that may be linked to, or accessed through, an article. The authors have granted ASM a non-exclusive, world-wide license to publish the Supplemental Material files. Please contact the corresponding author directly for reuse.
