# Supplementary material for: Pseudomonas fuscovaginae quorum sensing studies: 5% dominates cell-to-cell conversations
Source: Microbiol Spectr. 2024 Mar 21;12(5):e04179-23. doi: 10.1128/spectrum.04179-23 (PMC11064508; doi:10.1128/spectrum.04179-23)
Supplement: Supplemental material — Figures S1 to S5 and Videos S1 to S8. [file spectrum.04179-23-s0001.docx]

# SUPPLEMENTAL MATERIAL

##
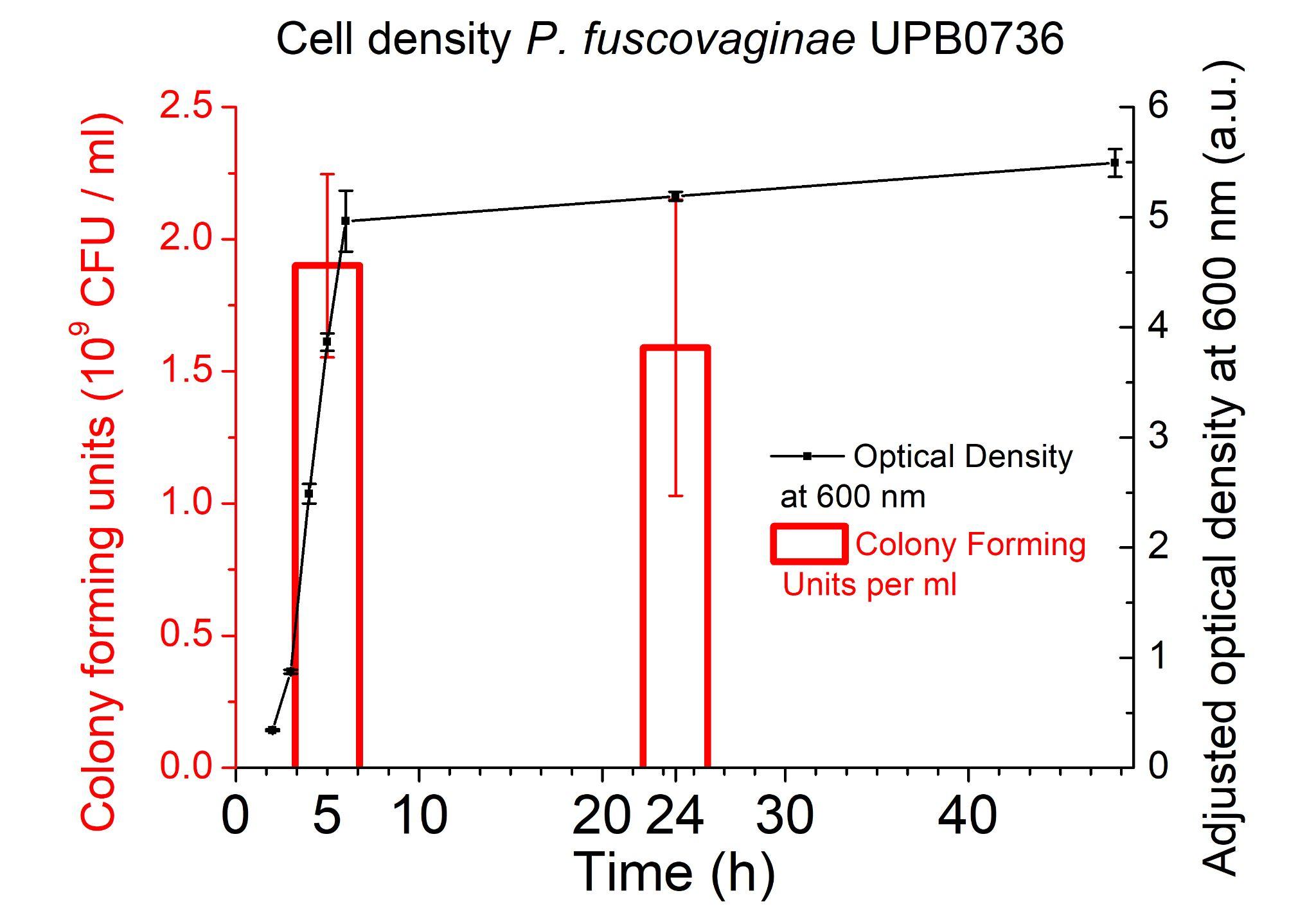
Figure S1: *P. fuscovaginae* UBP0736 growth dynamics

Growth curve measured by optical density at 600 nm and colony forming units per mL of *Pseudomonas fuscovaginae* UPB0736 wild type grown in King’s B (KB) medium at 30°C with shaking. The Colony forming units are shown on the primary y-axis (red; left) and the adjusted optical density at 600 nm is shown on the secondary y-axis (black; right). Averages of three biological replicates with standard error of means are shown.


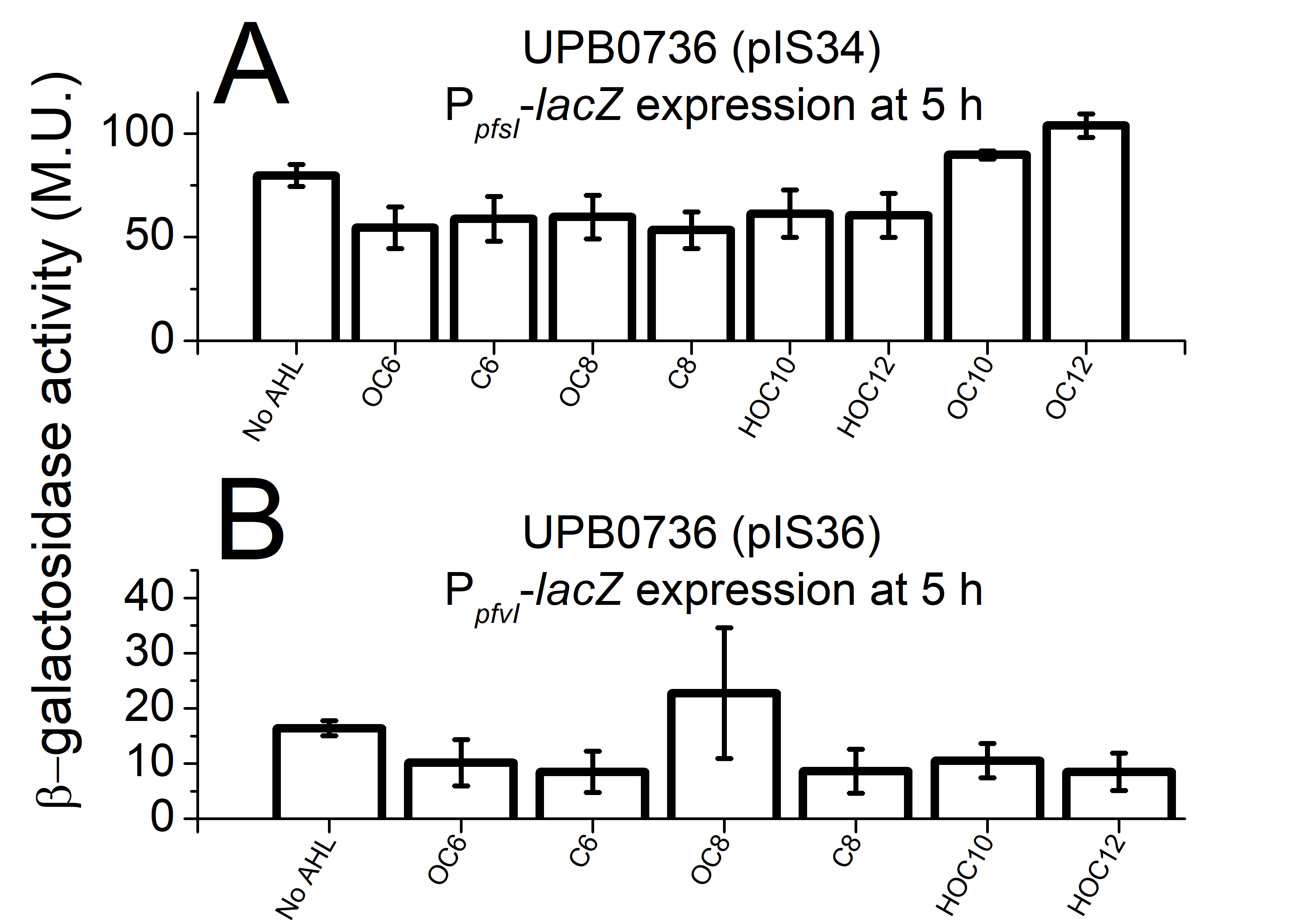


## Figure S2: AHLs that do not significantly increase the average QS response

Beta-galactosidase assay response after a 5 hrs incubation with 2.5 μM AHL signals of **A)** UPB0736 (pIS34) and **B)** UPB0736 (pIS36). Averages with the standard error of means of at least 3 biological replicates are shown. None of the values are significantly higher compared to the No AHL control where no exogenous AHLs were added (Two-sample t-test; P ≤ 0.05). 3-oxo-hexanoyl-L-Homoserine-lactone (OC6), hexanoyl-L-Homoserine lactone (C6), 3-oxo-octanoyl-L-Homoserine lactone (OC8), octanoyl-L-Homoserine lactone (C8), 3-hydroxy-decanoyl-L-Homoserine lactone (HOC10), 3-hydroxy-dodecanoyl-Homoserine lactone (HOC12), 3-oxo-decanoyl-L-Homoserine lactone (OC10) and 3-oxo-dodecanoyl-Homoserine lactone (OC12) did not elicit a significant response on **panel A**. OC6, C6, OC8, and C8, HOC10 and HOC12 did not elicit a significant response on **panel B.**


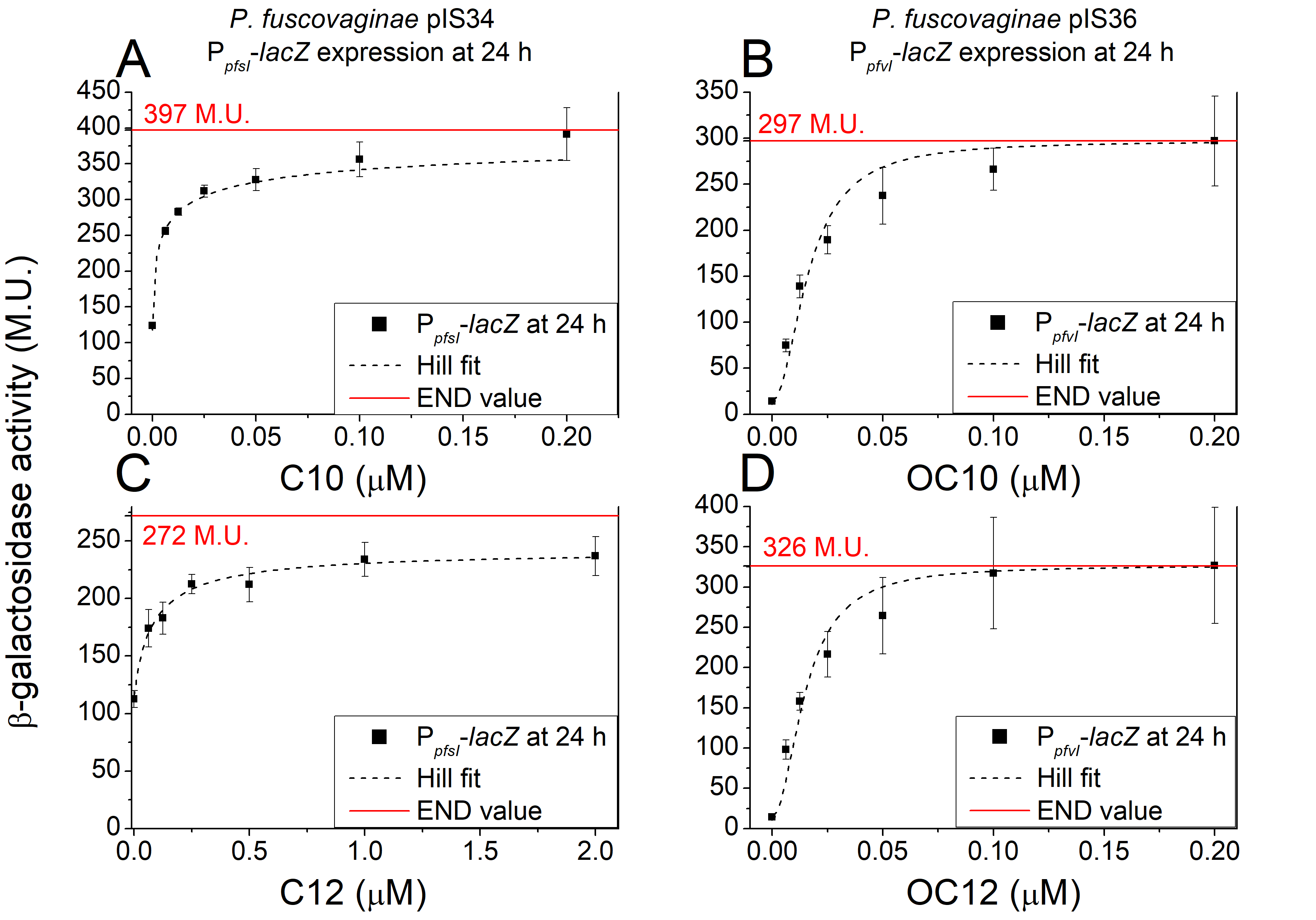


## Figure S3: AHL dose-response of *P. fuscovaginae* QS at 24 h

AHL Dose-response of *P. fuscovaginae* UPB0736 (pIS36) and UPB0736 (pIS36). The beta-galactosidase activity of different transcriptional reporter strains after 24 h of incubation with exogenous AHLs is shown in all panels. Data points indicate averages with error bars showing the standard error of means of at least 3 biological replicates. The dashed lines indicate a fitted Hill function (Hill1; OriginPro 8.5). The red line indicates the saturation value (END) of the Hill fit function. **A)** C10 AHL dose-response of UPB0736 (pIS36) **B)** OC10 AHL dose-response of UPB0736 (pIS36). **C)** C12 AHL dose-response of UPB0736 (pIS36) and **D)** OC12 AHL dose-response of UPB0736 (pIS36).


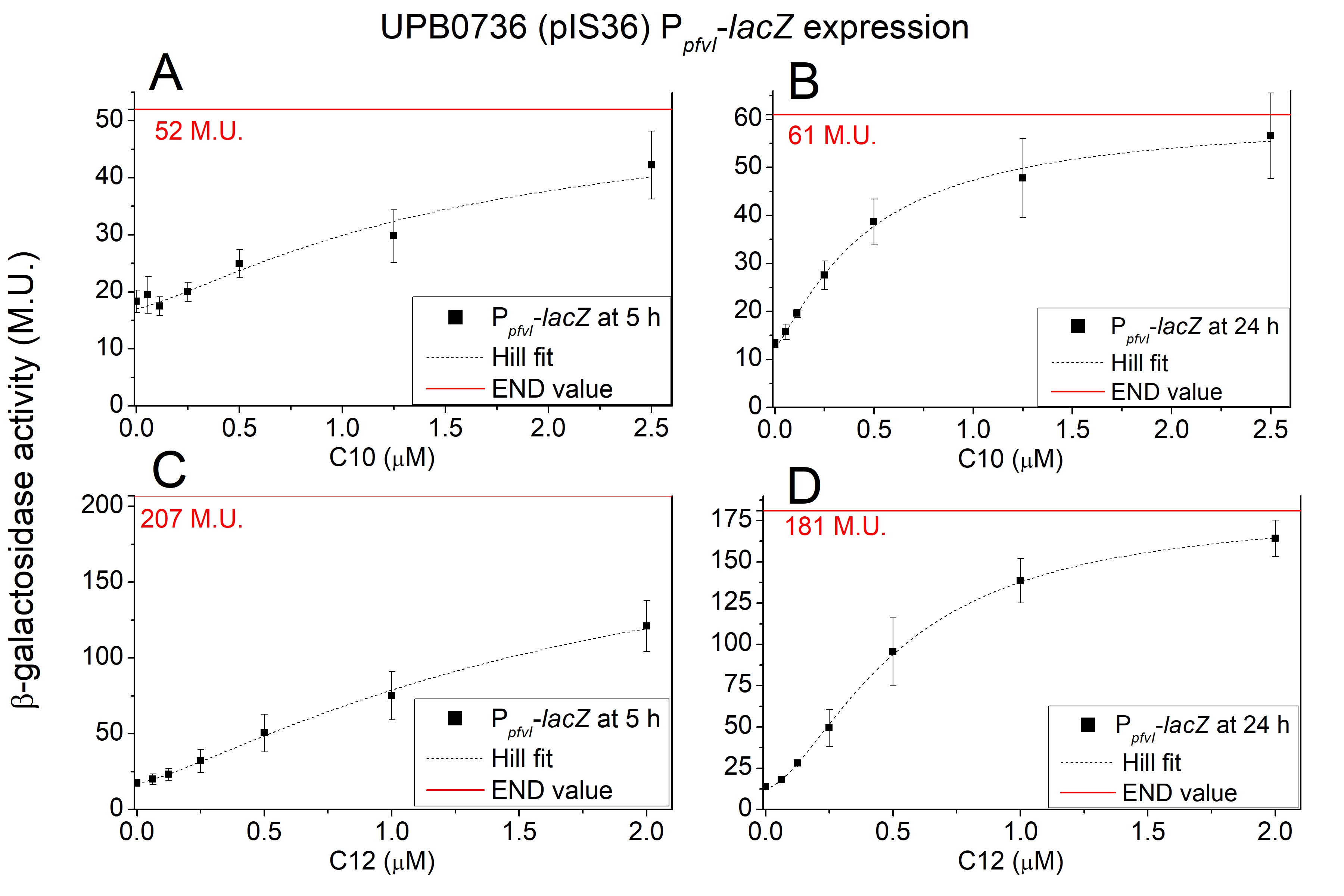


## Figure S4: AHL P*_pfvI_* transcriptional response saturation of *P. fuscovaginae* as betagalactosidase activity at 5 and 24 h

AHL Dose-response of UPB0736 (pIS36)*.* The betagalactosidase activity after incubation with exogenous AHLs is shown. Data points indicate averages with error bars showing the standard error of means of at least 3 biological replicates. The dashed lines indicate a fitted Hill function (Hill1; OriginPro 8.5). The red line indicates the saturation value (END) of the Hill fit function. **A)** C10 AHL dose-response relationship of UPB0736 (pIS36) after 5 hrs of incubation **B)** C10 AHL dose-response relationship of UPB0736 (pIS36) after 24 hrs of incubation **C)** C12 AHL dose-response relationship of UPB0736 (pIS36) after 5 h of incubation and **D)** C12 AHL dose-response relationship of UPB0736 (pIS36) after 24 hrs of incubation.


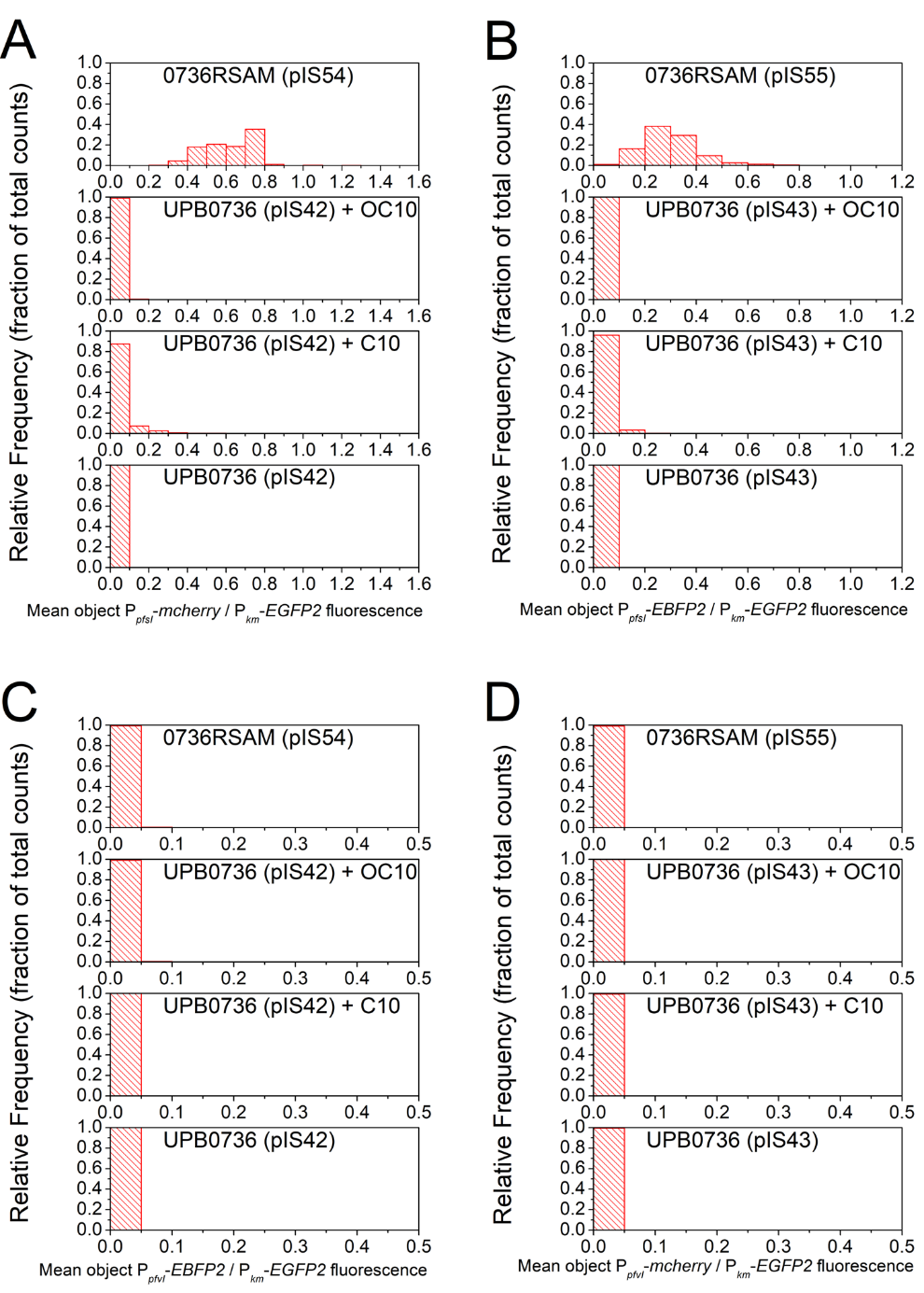


## Figure S5: Mean object fluorescence ratios of *P. fuscovaginae* transcriptional reporters

Fluorescent object counts of UPB0736 (pIS42), UPB0736 (pIS43), 0736RSAM (pIS54) and 0736RSAM (pIS55) A) Estimation of *pfsI* promoter activity with the red fluorescent transcriptional reporter constructs relative to constitutive green fluorescence. B) Estimation of *pfsI* promoter activity with the blue, fluorescent transcriptional reporter constructs relative to constitutive green fluorescence. C) Estimation of *pfvI* promoter activity with the red fluorescent transcriptional reporter constructs relative to constitutive green fluorescence. D) Estimation of *pfvI* promoter activity with the blue fluorescent transcriptional reporter constructs relative to constitutive green fluorescence.

## Video S1-S8: Growth of transcriptional reporters on solid media

Link to videos of *P. fuscovaginae* fluorescent transcriptional reporters UPB0736 (pIS42) and UPB0736 (pIS43) growing under agarose pads in 96-well media for 48 h. <https://www.youtube.com/playlist?list=PLRxYiWDI2LqQW79NH1ncEpHhXA75-hgvJ>
